# Muscle development in the shark *Scyliorhinus canicula*: implications for the evolution of the gnathostome head and paired appendage musculature

**DOI:** 10.1186/s12983-017-0216-y

**Published:** 2017-06-21

**Authors:** Janine M. Ziermann, Renata Freitas, Rui Diogo

**Affiliations:** 1 Department of Anatomy, Howard University College of Medicine, 520 W St NW, Washington, DC 20059 USA; 2 IBMC—Institute for Molecular and Cell Biology, Oporto, Portugal; 3 I3S, Institute for Innovation and Health Research, University of Oporto, Oporto, Portugal; 4 Department of Anatomy, Howard University College of Medicine, Washington, DC 20059 USA

**Keywords:** Muscles, Cranial, *Cucullaris*, Head, Pectoral, Pelvic, Fin, Shark, Limb

## Abstract

**Background:**

The origin of jawed vertebrates was marked by profound reconfigurations of the skeleton and muscles of the head and by the acquisition of two sets of paired appendages. Extant cartilaginous fish retained numerous plesiomorphic characters of jawed vertebrates, which include several aspects of their musculature. Therefore, myogenic studies on sharks are essential in yielding clues on the developmental processes involved in the origin of the muscular anatomy.

**Results:**

Here we provide a detailed description of the development of specific muscular units integrating the cephalic and appendicular musculature of the shark model, *Scyliorhinus canicula.* In addition, we analyze the muscle development across gnathostomes by comparing the developmental onset of muscle groups in distinct taxa. Our data reveal that appendicular myogenesis occurs earlier in the pectoral than in the pelvic appendages. Additionally, the pectoral musculature includes muscles that have their primordial developmental origin in the head. This culminates in a tight muscular connection between the pectoral girdle and the cranium, which founds no parallel in the pelvic fins. Moreover, we identified a lateral to ventral pattern of formation of the cephalic muscles, that has been equally documented in osteichthyans but, in contrast with these gnathostomes, the hyoid muscles develop earlier than mandibular muscle in *S. canicula*.

**Conclusion:**

Our analyses reveal considerable differences in the formation of the pectoral and pelvic musculatures in *S. canicula*, reinforcing the idea that head tissues have contributed to the formation of the pectoral appendages in the common ancestor of extant gnathostomes. In addition, temporal differences in the formation of some cranial muscles between chondrichthyans and osteichthyans might support the hypothesis that the similarity between the musculature of the mandibular arch and of the other pharyngeal arches represents a derived feature of jawed vertebrates.

## Background

The origin of jawed vertebrates (gnathostomes) is undoubtedly one of the major events in the history of life, as it drastically changed the feeding modes among vertebrates [1]. The origin of jaws, paired fins, and the cephalic and appendicular musculature was probably of chief importance for the transition from suspension feeding to predation in this particular vertebrate lineage [1]. These novel morphological features may also have contributed to the vast radiation of gnathostomes, which make up more than 99.9% of all living vertebrates [2]. Chondrichthyans such as the sharks are considered to have morphological characteristics that retained various plesiomorphic gnathostome traits [3, 4]. These cartilaginous fishes have been around for over 400 million years and are, therefore, amongst the oldest surviving vertebrate groups [5]. Although they have unique features that evolved in ways distinct from other fishes, they possess, for example, the most plesiomorphic paired fin structure of modern vertebrates [5]. Therefore, sharks are also relevant for discussions on the development and evolution of not only fish muscles but also of the muscles of vertebrates as a whole [6]. Moreover, striking similarities were detected between the musculature of chondrichthyans and placoderms, which are fossil representatives of the most basal gnathostomes [7, 8]. This makes them ideal extant models to study the evolution of paired (i.e., pectoral and pelvic) appendage musculature, providing a unique opportunity to investigate the developmental processes involved in the formation of these tissues during early evolution of gnathostomes [9–11]. Sharks, in addition, belong to the living sister group of osteichthyans (bony fish + tetrapods), where developmental studies are mostly performed, which makes them fundamental models for phylogenetic sampling (e.g., [10–12]). Data from cyclostomes - which are the only extant representatives of agnathans - such as the lamprey are also extremely relevant to understand the origin and early evolution of gnathostome morphology [13–20].

Comparative analyses have provided descriptions of the musculature of the head, neck, and locomotory appendages across various vertebrate lineages [18, 21–26]. However, little information is available on how and when each of the specific muscles develop in organisms that may have retained plesiomorphic gnathostome features, as with the shark. Currie and colleagues used shark models to investigate how the mechanisms that generate appendicular muscles evolved [12, 27]. These authors confirmed the observations obtained in studies carried out at the end of the nineteenth century showing that, in sharks, the appendicular musculature is formed by epithelial somitic extensions that penetrate the fin buds during development. However, their work was not focused on providing a detailed description on the development of specific muscles, such as those that connect the pectoral appendages to the skull. The only studies providing details on the development of individual muscles of sharks were published several decades ago (e.g., cephalic musculature reviewed by Edgeworth [23]), but they lack validation with novel methodological approaches. Therefore, detailed studies on shark muscle development are required to further explore potential ancient developmental processes involved in the formation of the cephalic and appendicular musculature in gnathostomes.

Several studies suggest a striking conservation of the developmental patterning of cephalic muscles, which is particularly well documented in amphibians [28–31]. These include the observation that these muscles tend to differentiate from the anterior to the posterior. For example, mandibular and hyoid muscles normally appear earlier than the muscles of the branchial (i.e., the most posterior pharyngeal) arches. Their development also tends to follow a lateral to medial direction. For instance, lateral muscles of one arch tend to differentiate earlier than the more medially and ventral muscles of the same arch. In addition, these muscles normally develop from their region of origin towards their region of insertion [28, 32, 33]. It remains unknown, however, if this temporal and spatial sequence of developmental events represents the plesiomorphic feature, present in the common ancestor of all gnathostomes. Diogo and colleagues suggested that, in general, the developmental order of appearance of the cephalic muscles of amphibians [28, 29] and zebrafish [34] parallels the evolutionary order of appearance. This is also the case for the cephalic muscles in the head, neck, and limb muscles of primates [35]. Developmental studies on sharks are crucial to investigate if such patterns are also seen in chondrichthyans, exploring the conservation of these developmental patterns within gnathostomes, and whether there is a parallelism between ontogeny and phylogeny in vertebrate muscle development in general.

Within the broader analysis of cephalic muscle development in vertebrates, special attention has been given to the puzzling muscle *cucullaris*, which is deeply related to one of the most crucial evolutionary events during vertebrate evolutionary history: the evolution of the neck [36]. Recently, Ziermann and colleagues proposed that neck evolution was a long, stepwise macroevolutionary event, involving a stage in which an undivided *cucullaris* was connected to the branchial arches and the pectoral girdles [18], followed by its subdivision into the *levatores arcuum branchialium* attaching to the branchial arches and the *protractor pectoralis* attaching to the pectoral girdle, in osteichthyans [18]. Subsequently, there was further differentiation of the *protractor pectoralis* into various muscles (e.g., *trapezius*, *sternocleidomastoideus*) that took place during the evolution of tetrapods, where the head became further separated from the trunk [18]. Remarkably, recent analyses of the musculature in placoderm fossils suggest that the *cucullaris* might not have attached to the pectoral girdle in at least some of the members of this extinct group [36]. The comparison of the osteichthyans developmental data with the information obtained via the detailed analyses of the development of the *cucullaris* in chondrichthyans may offer additional information to discuss the ancestral condition of the *cucullaris* and thus the evolution of the neck within gnathostomes.

Additionally, comprehensive myological analyses in shark embryos may also help elucidate how paired appendage musculature was acquired within the gnathostome lineage. Two influential hypotheses were proposed during the late nineteenth century to explain the origin of two sets of paired appendages (pectoral and pelvic) in vertebrates: the gill - arch theory and the lateral fin-fold theory. The gill-arch theory proposes that pectoral and pelvic appendages evolved from modified gill arches and the pelvic appendages secondarily migrated caudally [37], whereas the lateral fin-fold theory suggests that pectoral and pelvic fins derive from a hypothetical bilateral continuous embryonic finfold. Both theories are consistent with the hypothesis that pectoral and pelvic appendages are serial homologous [38–40]. However, within these two theories the fin-fold theory, which lacks paleontological evidences [41, 42], excludes the contribution of head tissues to the formation of pectoral appendages. When molecular analyzes became available, the involvement of a common set of molecular mechanisms activated within a continuous dorsal/ventral field of competence to form appendages (“competent stripe”, [13]) during the development of not only paired but also unpaired appendages were consistent with this idea that all these appendages do share similar developmental mechanisms [9–11, 43–46]. However, Gillis and colleagues, have shown that there are remarkable similarities in the developmental mechanisms operating during the ontogeny of the branchial arches and pectoral fin development [47, 48], thus reigniting discussions on Gegenbaur’s hypothesis.

It is worth noting that the theories regarding the origin of two sets of paired appendages in gnathostomes mainly target the initial developmental components of fins, which are the fin mesenchyme and the endoskeleton that differentiates within and from it. However, dismissed from these theories are additional components essential for fin/limb function and which probably reinforced their adaptive rate, such as muscles, nerves, or blood vessels. Comparative myogenic studies performed by Diogo and colleagues, integrated with data from other authors and fields, suggest that the musculature of pectoral (fore-) and pelvic (hind-) appendages are particularly different in the proximal (girdle) region of these appendages [22, 49, 50]. These data question the existence of a common serial homologue musculature in pectoral and pelvic appendages, and indicate that what makes the pectoral and pelvic appendages so unique, and so remarkably similar in derived gnathostomes such as tetrapods, might in fact be the result of derived co-option [51]. To gain insight into these questions, it is crucial to comparatively evaluate muscle development in the pectoral and pelvic appendages in animals retaining a plesiomorphic fin musculature within gnathostomes, the sharks, and evaluate the contribution of cranial muscles to appendicular muscles during their formation.

Therefore, to discuss the broader developmental and evolutionary issues mentioned above, we present a detailed timeline of the development of both the cephalic and paired appendicular muscles in a shark species, *Scyliorhinus canicula*. We identified heterochronic events during the development of the cephalic muscles of the shark as compared to the developmental pattern reported for most osteichthyans. That is, in our analyses of the shark the hyoid muscles develop earlier than mandibular muscles. This pattern contrasts with the observations in most osteichthyans where usually the mandibular muscles develop before the hyoid muscles or both groups develop simultaneously. In addition, we found that, although the development of the pectoral and pelvic appendicular muscles share similarities, there are significant differences concerning the timing of their formation. Moreover, tight muscular connections, involving several muscular units, develop between the pectoral girdle and the cranium of sharks, which finds no parallel during myogenesis of the pelvic fins. Our results highlight the importance to trace the distinct evolutionary processes analyzing different tissues individually and making use of model organisms at key phylogenetic positions.

## Methods

### Collection and staging of embryos

*Scyliorhinus canicula* (L. 1758) eggs were collected from the Menai Strait (North Wales). Embryos were isolated from egg cases and dissected from the yolk sac in ice-cold phosphate-buffered saline (PBS). Specimens were then staged according to Ballard et al. [52], before being fixed and processed as described below.

### SEM and histology

For the scanning electron microscopy (SEM), specimens were fixed in 1% glutaraldehyde, then treated with 1% osmium tetroxide, dehydrated in a graded ethanol series, and transferred to acetone. Subsequently the specimens were critical-point dried, mounted onto carbon discs, sputter-coated with gold particles and visualized in a Jeol JSM-T300 Scanning Electron Microscope. For histology, embryos were fixed in 4% paraformaldehyde, dehydrated in a graded ethanol series, washed in Xylene, and embedded in paraffin. The resultant microtome sections (10 μm) were stained using Mallory’s Triple Stain.

### Whole-mount immunochemistry

*S. canicula* muscle development was characterized in the embryonic time comprising stages 23 to 32 using immunochemistry with antibody against Myosin Heavy Chain (MyHC; A4–1025, DSHB), a marker of muscle differentiation [12], following previously established and described protocols [53]. We analyzed one embryo per stage for muscle development using immunochemistry, as our previous muscle developmental studies have indicated that the intraspecific variability concerning the timing of muscle development depends also on sampling density and here the stages were clearly separated [54].

Regarding immunochemistry, the specimens were fixed in 4% PFA, then washed in PBS with 1% triton (PBT-1) for 3 h, incubated in 0.25% trypsin for 2–5 min, rinsed in PBT-1, and immersed in pre-cooled acetone for 10 min. After a brief rinse in PBT-1, the embryos were placed in blocking solution containing 10% goat serum (GS), 1% dimethyl sulfoxide, and 5% H2O2 in PBT-1, overnight. The MyHC antibody was used in a concentration of 1:10 and was diluted in PBT-1 containing 10% GS. Goat anti-mouse IgG secondary antibody, HRP (Thermofisher), was used at a concentration of 1:500 diluted in PBT-1 with 1% GS. Embryos were then washed in 1% GS in PBT-1, followed by PBS, and then incubated in 0.5 mg/ml diaminobenzidine (DAB). The reaction was developed by transferring embryos to fresh DAB activated with 0.003% H2O2.

### Muscle characterizations

One side of the embryos was dissected with micro-dissection tools under a dissection microscope to analyze the development of deep muscles. The specimens were photographed at a dissecting microscope (Nikon SMZ-2B) equipped with a Nikon DS Fi1 5 Megapixel Color Camera Head. Myological terminology used in the present paper follows that proposed by Diogo and Abdala [21] and updated by Ziermann et al. [18] and Diogo and Ziermann [22] for adult sharks.

## Results

### Pectoral fin development and muscle differentiation

**Fig. 1:**
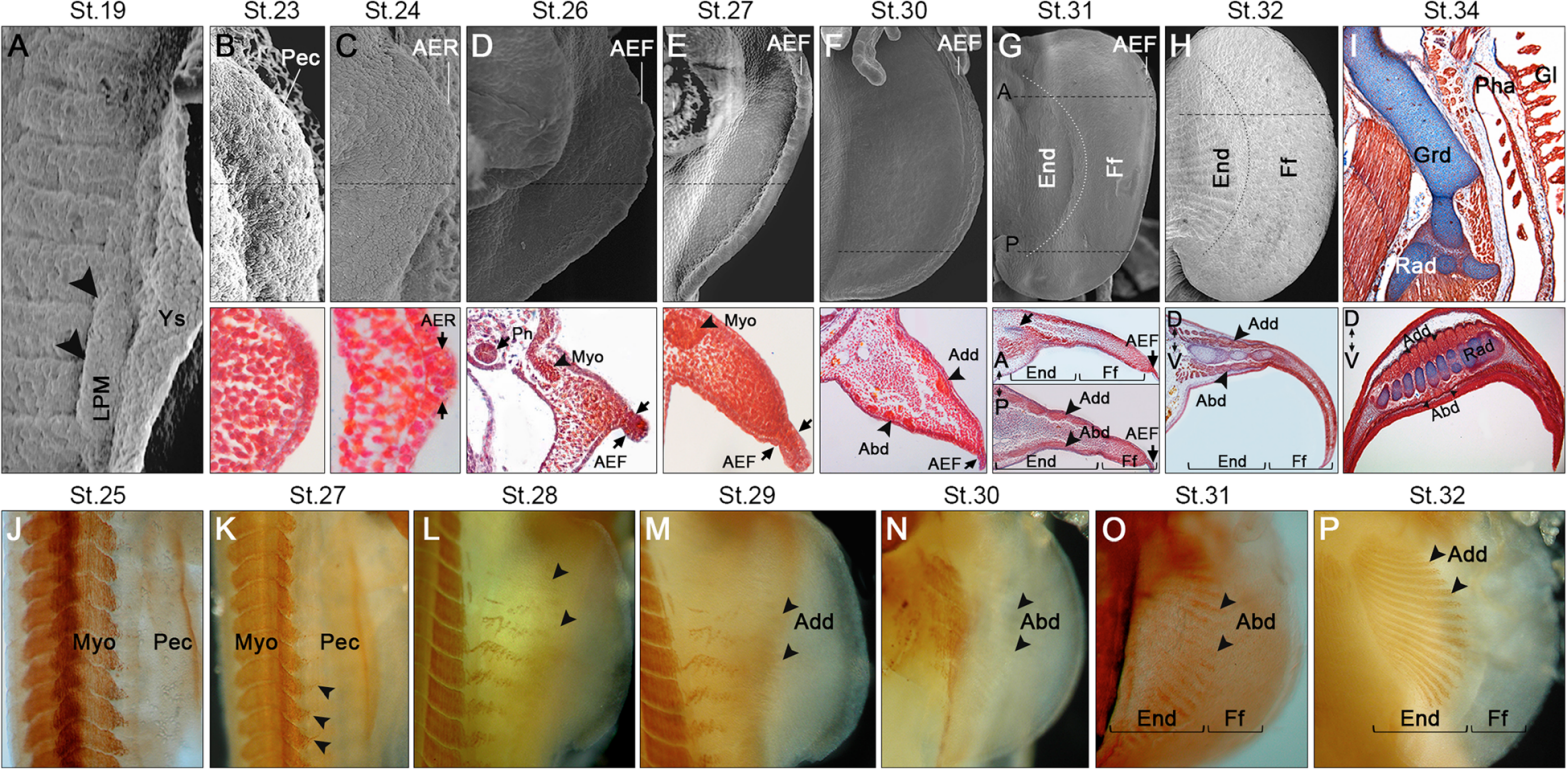
Pectoral fin development in *Scyliorhinus canicula.* Developmental stages (St.) at the *top*. **a** Scan electron microscopy (SEM) showing initial outgrowth of the lateral plate mesoderm (*arrowheads*), in the ventrolateral region between somites 6 and 16 (S6; S16), which will give rise to the pectoral fins. **b-i** SEM in *upper* panels (anterior to top) and Mallory’s trichrome stains of histological transversal sections in *lower* panels (dorsal to top). *Dashed lines* in *upper* panels indicate approximate plane of section shown in *lower* panels. **b** Initial pectoral fin buds (Pec) prior to the formation of the apical ectodermal ridge (AER). **c** Formation of the AER in the most distal ectoderm. **d-e** Formation of the apical ectodermal fold (AEF) by convergence of dorsal and ventral ectodermal cells at the distal fin tip. Note dermomyotome projections (Myo) starting to enter the pectoral fin buds. **f** Expansion of the *abductor superficialis* (Abd) and *adductor superficialis* (Add) muscles, ventrally and dorsally, respectively. **g** Pectoral fin with two identifiable domains: a proximal domain in which the endoskeleton elements differentiate (End) and a distal finfold (Ff), filled with mesenchymal cells and still capped with an AEF at this stage. Note: first chondrogenic condensations in the anterior part of the fins marked in *blue* (*arrow*) and *abductor* and *adductor* muscles in all presumptive End region. **h** Chondrogenic condensations in the End region, prominent finfold and AEF undetectable. Prominent *abductor superficialis* and *adductor superficialis* muscles covering the radials (Rad). **i** Shoulder girdle (Grd) forms parallel to the pharyngeal arches (Pha) that sustain the gills (Gl). Chondrogenesis of the radials (Rad) is close to completion as is development of the *abductor superficialis* and *adductor superficialis* muscles. **j-p** MyHC antibody stains throughout pectoral fin development. Dorsal views in J, K, L, M and P and ventral views in N and O. Note muscle projection expanding ventrally towards the fin field at stage 27 and entering the fin field by stage 28 (arrows). Adductor muscles are detected earlier (stage 29) than abductor muscles (stage 30). *Adductor superficialis* muscles are detected with MyHC stain in the entire endoskeleton domain between stages 31 and 32

**Fig. 2:**
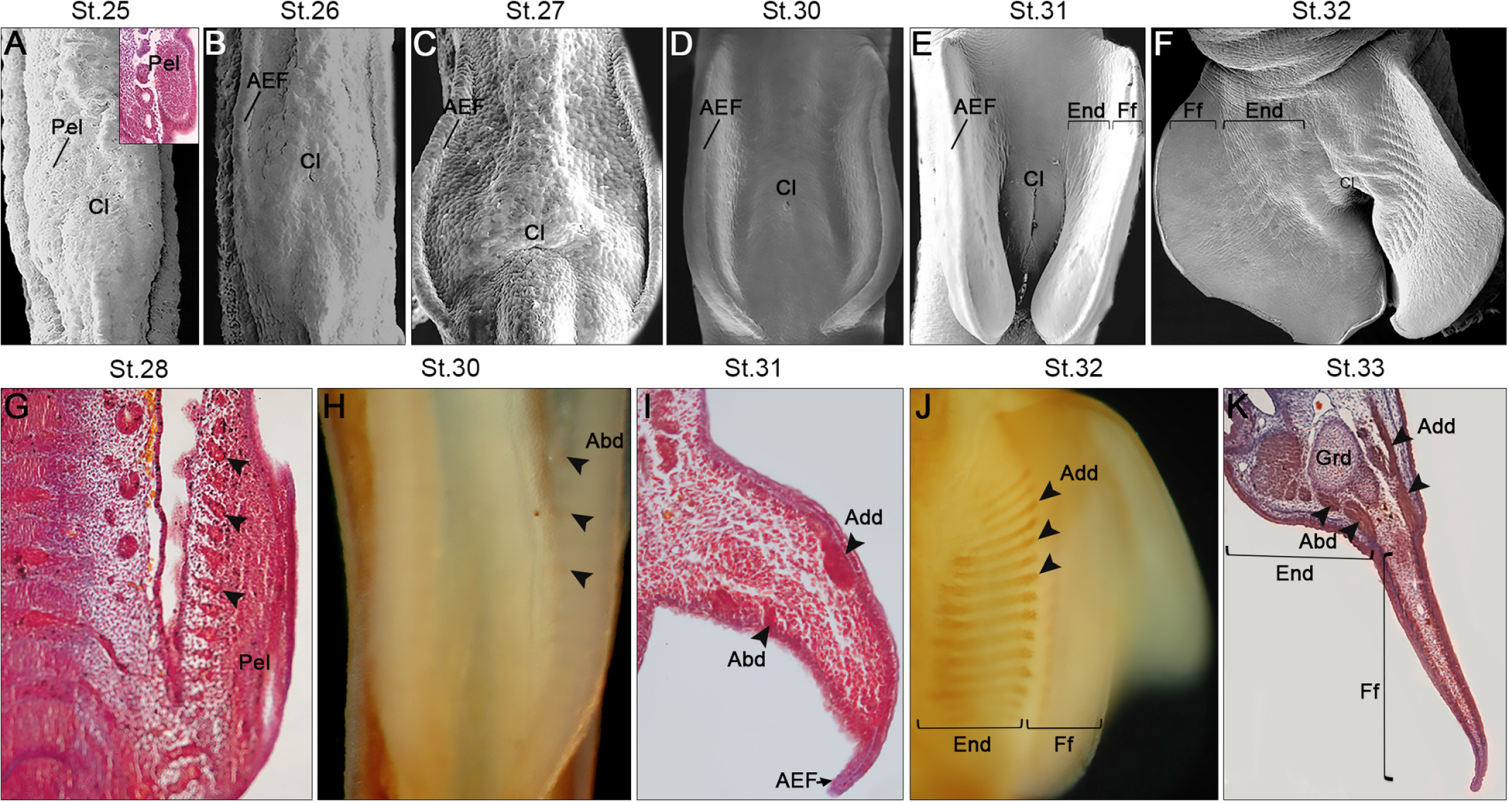
Pelvic fin development in *Scyliorhinus canicula.* Developmental stages (St.) at the *top*. **a** Scan electron microscopy (SEM) and Mallory’s trichrome stains of histological transversal sections showing initial pelvic fin outgrowth (Pel), laterally to the prospective cloaca region (Cl). **b-f** SEMs showing the progression of pelvic fin development (ventral views). Note formation of the apical ectodermal fold (AEF) at stage 26 and separation of a proximal domain in which the endoskeleton differentiates (End) and a distal domain, the finfold (Ff) by stage 32. **g-k** Muscle development between stages 28 and 33 shown with Mallory’s trichrome stains (**g**, **i**, **k**) and MyHC antibody stain (H,J). **g** Muscle projections entering the fin territory (*arrows*). **h** Abductor muscles observed ventrally (Abd). **i** Abductor and adductor superficialis (Add) muscles detected ventrally and dorsally, respectively. **j** Dorsal view showing adductor muscles detected from the proximal part of the fin to the distal border of the endoskeleton domain (End), but not in the finfold (Ff). **k** Chondrogenesis is close to completion, with the pelvic girdle (Grd) clearly detected proximally and prominent abductor and adductor muscles

**Fig. 3:**
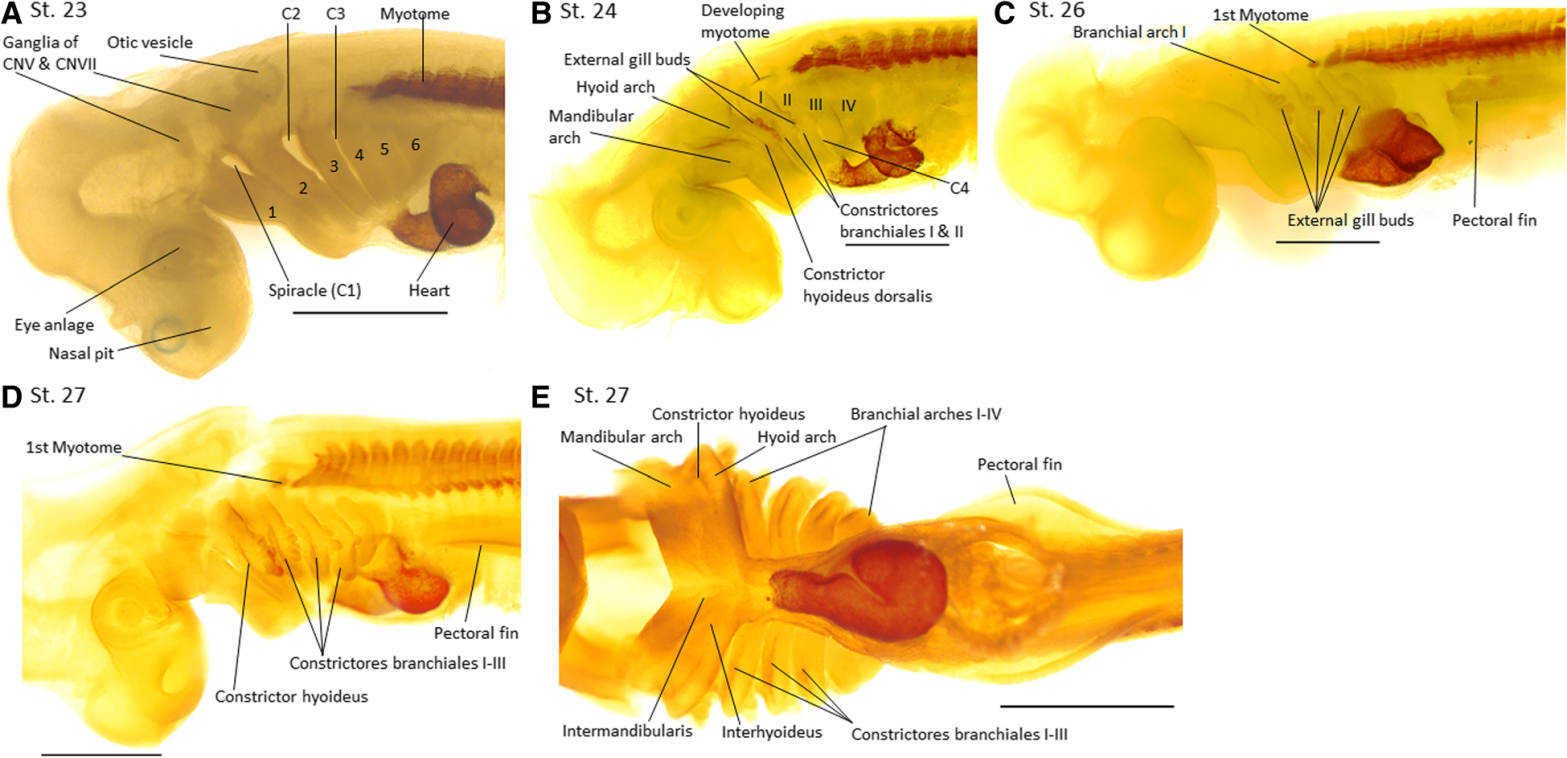
MyHC expression during muscle differentiation between stages 23 and 27 in *Scyliorhinus canicula*. In all figures anterior (or cranial) is towards the *left*. **a**-**d** left lateral views; **e** ventral view. **a** Stage 23; six pharyngeal arches (1–6) and the first three pharyngeal clefts (C1–3, opening of the pouches 1–3) are visible. The first pharyngeal arch is the mandibular arch, the second is the hyoid arch and the following four are branchial arches I-IV. **b** Stage 24; first cephalic muscle anlagen are indicated by faint staining laterally in the hyoid arch (*constrictor hyoideus dorsalis*) and the first three branchial arches (*constrictores branchiales I-III*); the opening of the fourth pharyngeal pouch (C4) is visible; first external gill buds appear. **c** Stage 26; the pectoral fin bud is clearly developed. **d-e** Stage 27. **d** Muscle development is clear in the hyoid arch (*constrictor hyoideus dorsalis*) and branchial arches I to III (*constrictores branchiales I-III*). **e** First muscle staining appears ventrally in the mandibular arch. Scale bar = 1 mm

**Fig. 4:**
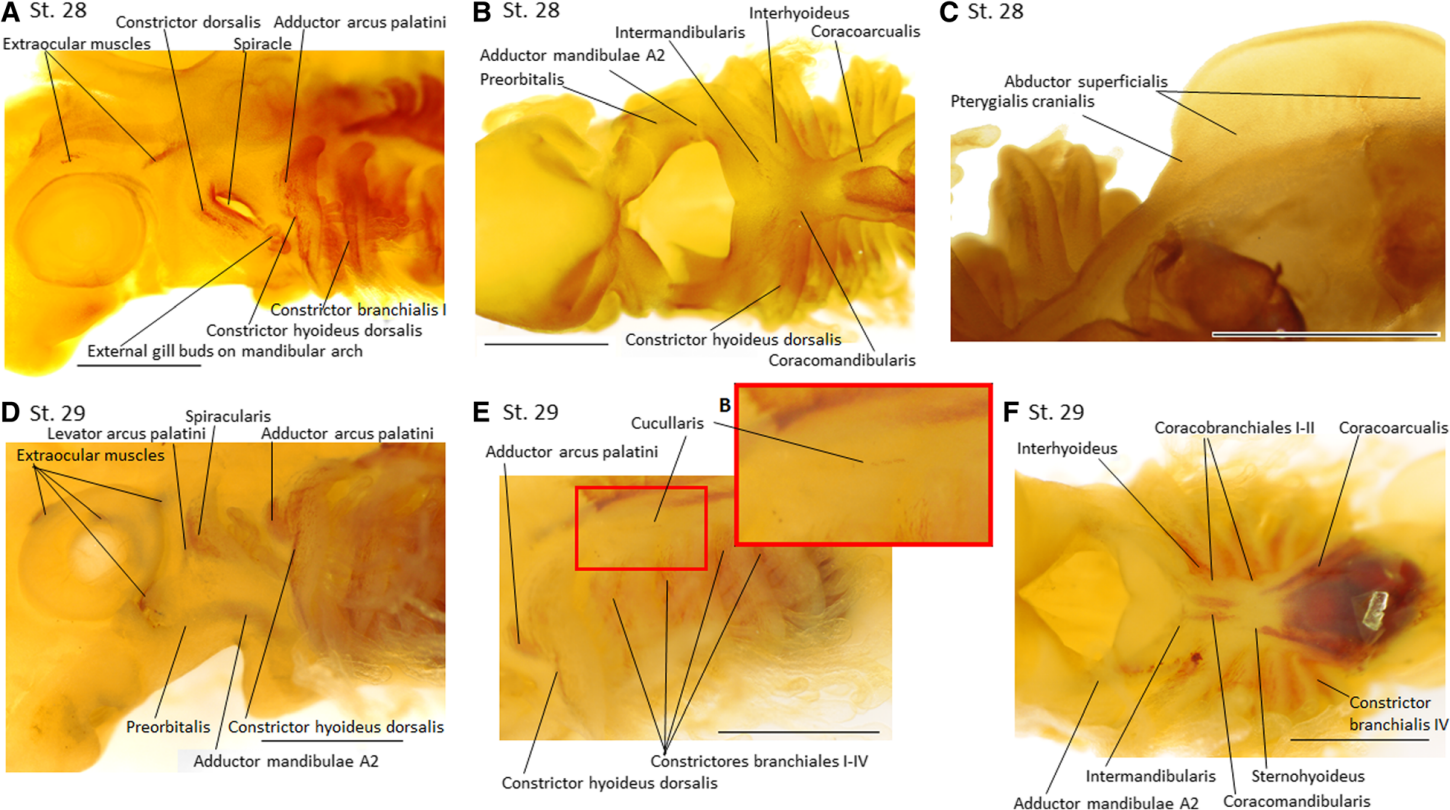
MyHC expression during muscle differentiation between stages 28 and 29 in *Scyliorhinus canicula*. Cranial to the *left*. **a-c** Stage 28; **a** While the dorsal constrictor of the mandibular arch is just now appearing, the dorsal constrictor of the hyoid arch is already diving into the adductor arcus palatini and the *constrictor hyoideus dorsalis*. **b** The ventral muscles of the mandibular and hyoid arch are all present, while the more posterior ventral branchial muscles are still not developed. **c** Ventral view of the pectoral fin with the first fin muscle appearing. **d-f** Stage 29; **d** The dorsal constrictor of the mandibular arch divides into the spiracularis and the levator arcus palatini. **e** Enlarged window to show the faint cucullaris staining. **f** Ventral branchial and hypobranchial muscles further differentiated. Scale bar = 1 mm

**Fig. 5:**
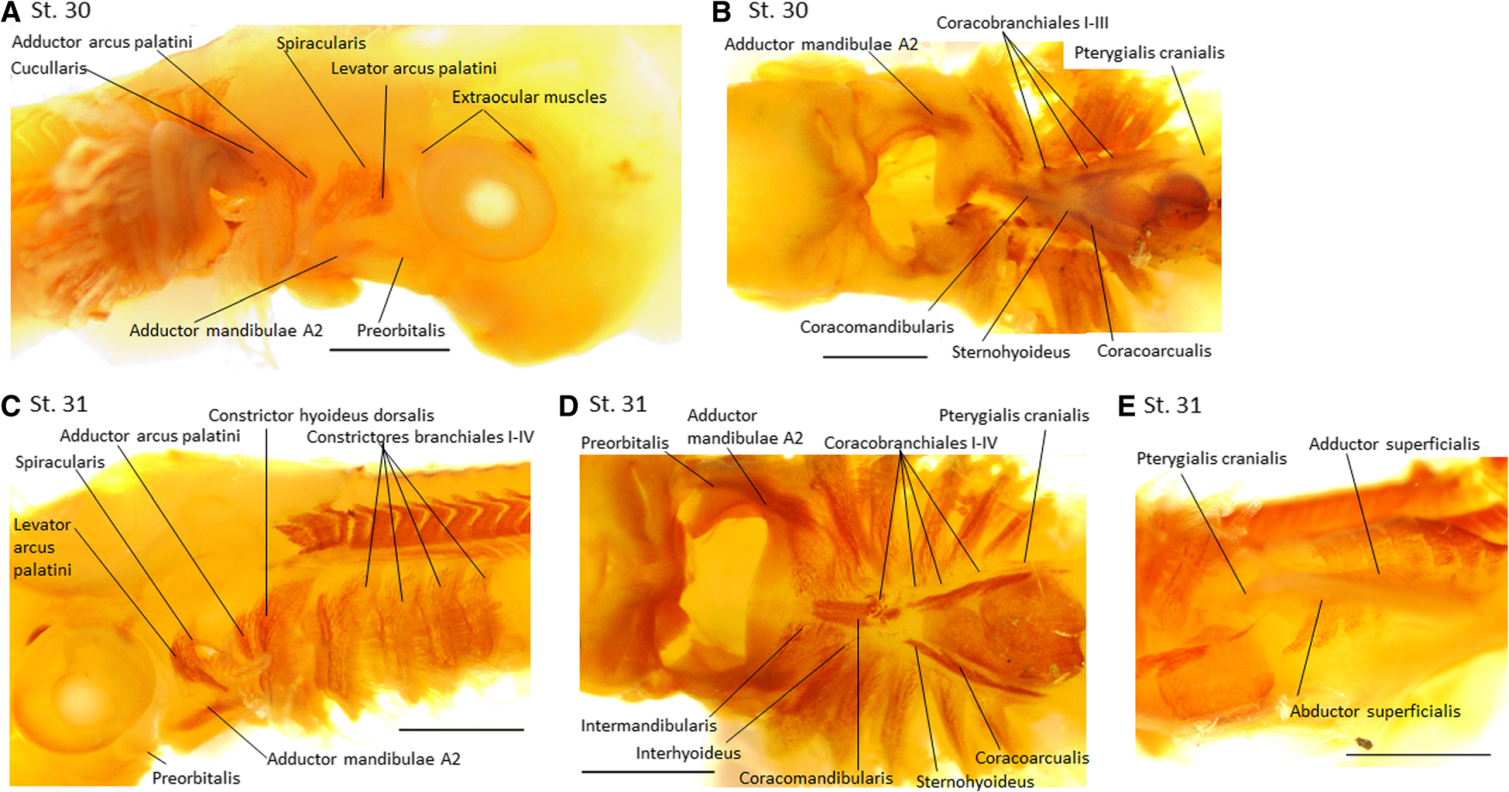
MyHC expression during muscle differentiation between stages 30 and 31 in *Scyliorhinus canicula*. **a**, Cranial to the *right*; **b**-**e**, Cranial to the *left*. **a-b** Stage 30; **a** Spiracularis and levator arcus palatini start to separate, while the dorsal muscles of the hyoid arch are widely separated. The cucullaris extends, but does not attach to branchial arches. **b** Branchial and hypobranchial muscles further differentiated. **c-e** Stage 31; **c** Mandibular and hyoid arch muscles become better distinguishable. **d** Ventrally the intermandibularis and interhyoideus come into contact and four coracobranchiales are now visible. **e** In the pectoral fin, the adductor superficialis and abductor superficialis are clearly visible. Scale bar = 1 mm

**Fig. 6:**
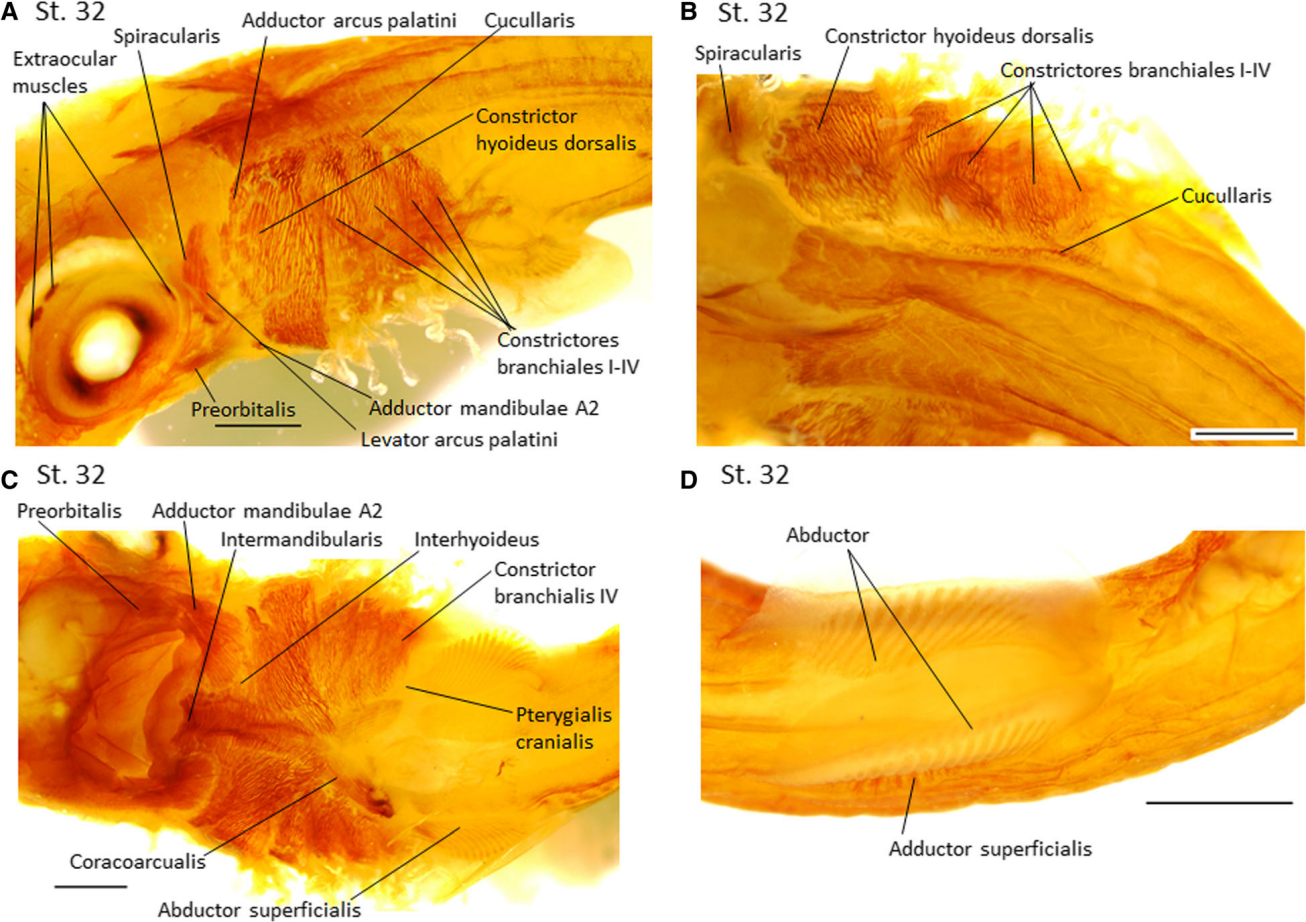
MyHC expression during muscle differentiation at stage 32 in *Scyliorhinus canicula*; cranial to the *left*. **a** The *constrictores branchiales I-IV* completely cover the branchial arches; deep branchial muscles cannot be identified. **b** The cucullaris muscle stretches dorsally to the branchial arches with dorsally attaching to the most caudal one. **c** The ventral aspect is almost completely covered by muscles. Deep branchial muscles, such as the coracobranchiales, are no longer visible. **d** Ventral view of pectoral region. The abductor and the adductor superficialis are expanded. Scale *bar* = 1 mm

The development of the cephalic and appendicular musculature of *S. canicula* is summarized in Table 1 and shown in Figs. 1, 2, 3, 4, 5 and 6. Muscle projections are detected extending from the myotome towards the pectoral fin fields between **stages 26** and **27** (Fig. 1d-e; j-k). MyHC staining indicates that muscle projections invade the pectoral fin territory between **stages 28** and **29** (Fig. 1l-m). The former shows the development of the dorsal muscle *adductor superficialis* and the ventral muscle *abductor superficialis* (Figs. 1l-m and 3c-d). At **stage 30**, muscle projections are detected throughout the fin, both dorsally and ventrally (Fig. 1f). MyHC staining further indicates that the *abductor superficialis*, which connects the girdle to the fin, is now prominent at this stage (Fig. 1n). MyHC staining also highlights the rostroventral development of the *pterygialis cranialis* at **stage 28** (Fig. 4c). Between **stages 31** and **32**, the *adductor superficialis* and *abductor superficialis* pursue development (Figs. 1g-h, o-p; 6d), and the *pterygialis cranialis* continues to expand and differentiate without major changes relative to the previous stages (Figs. 5d, e and 6c). Finally, at **stage 34 and** prior to hatching, the appendicular skeleton appears strongly associated to the musculature both in the girdle and in the pectoral fins (Fig. 1i).

**Table 1:**
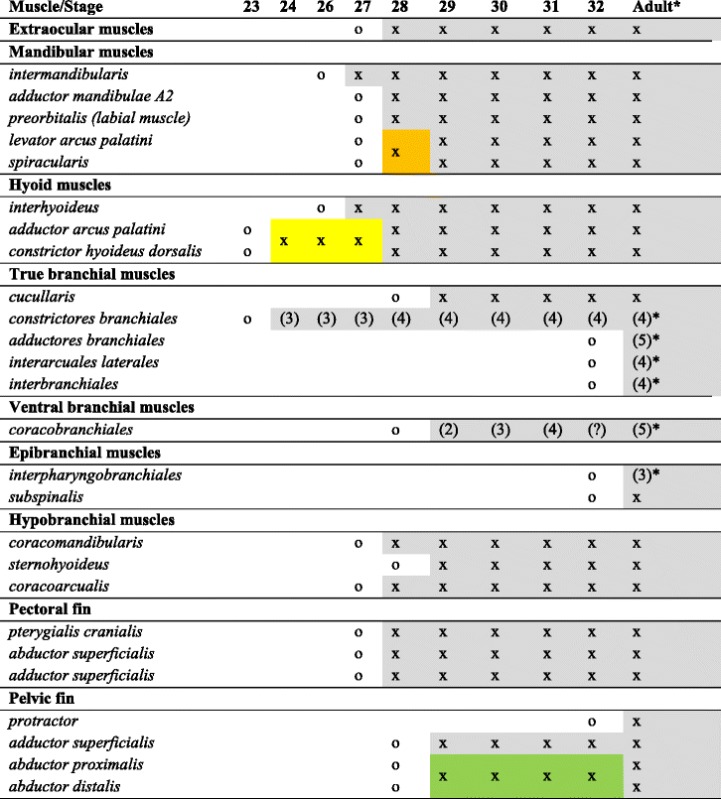
Myogenic cephalic and pectoral/pelvic fin development in *Scyliorhinus canicula*. Stages according to Ballard et al. [52]. Terminology and adult characteristics follows Ziermann et al. [18]. x = present (independent on status of differentiation); o = absent (or not stained); (number) = number of repetitive muscles (usually corresponding to branchial arches and counting from anterior to posterior). Orange box = dorsal constrictor of mandibular arch. Yellow box = *constrictor hyoideus.* Green box = only one abductor could be found in the *S. canicula* stages investigated here. (?) could not be observed because of overlying muscles. *Adult condition is from *Squalus acanthias* [18]

The first signs of pectoral fin development are detectable at **stage 19**, budding out from the lateral plate mesoderm and positioned approximately between somites 6 to 16 (Fig. 1a). By **stage 23**, the pectoral fin buds are visible lateral to the yolk stalk [53] (Fig. 1b). However, the apical ectodermal ridge (AER), a crucial signaling center during fin/limb development, is undetectable up to **stage 24** (Fig. 1c). Then, the pectoral fin buds re-shape dorsoventrally acquiring a disc-like structure capped distally by the AER (Fig. 1c). This distal structure is rapidly converted into an apical ectodermal fold [53] (AEF) by **stage 25** (Fig. 1d).

#### Pelvic fin development and muscle differentiation

The pelvic fins start to bud out from the lateral plate mesoderm laterally to the cloaca region at **stage 25** and their development is close to completion prior to hatching [9] (Fig. 2a-f). As for the pectoral appendages, the formation of the AER seems to be transient, rapidly giving rise to an AEF, at **stage 26** (Fig. 2b). As seen in the pectoral appendages, MyHC staining and histology suggest that muscle projections extend ventrally towards the base of the pelvic fins between **stage 27** and **stage 28** (Fig. 2c, g). At **stage 30** an undivided *abductor* and the *adductor superficialis* can be identified (Fig. 2d, h). However, pelvic fins show an even fainter staining on both the dorsal and ventral sides indicating just the initial development of the *adductor superficialis* and *abductor*, respectively (compare Fig. 1n with Fig. 2h). The latter is easier to see at **stage 31** (Fig. 2e, i). These muscles then expand along the proximodistal axis of the pelvic fins during stage 31 (Fig. 2e) reaching the margin of the terminal finfold by **stage 32** (Fig. 6d). While formation of the endoskeletal elements continues at **stage 33**, the distinction between a proximal abductor and a distal abductor, as well as the presence of a separate muscle protractor of the pelvic fin as described in adult sharks [22], cannot be made. As these are superficial structures, it is likely that they are not differentiated yet in any of the stages analyzed by us, which is plausible due to the fact that the pelvic fins start to form later than the pectoral fins.

#### Cranial development and muscle differentiation

The first signs of eye and nasal pit development are detected in *S. canicula* at **stage 17** and **21**, respectively (data not shown). As early as **stage 19**, features such as the otic vesicles and the curvatures delineating the forebrain, the midbrain, and the hindbrain are visible [10]. At **stage 23**, the ganglia of cranial nerves V and VII are clearly observable through the skin (Fig. 3a). Furthermore, six pharyngeal arches are present as anlagen and the proximity to the posteroventrally looping heart is clearly visible [52] (Fig. 3a). The first pharyngeal arch is the mandibular arch, the second is the hyoid arch, and the following four are branchial arches I-IV (i.e., pharyngeal arches III-VI). The first three pharyngeal pouches are open, with the first one being the spiracle (C1, hyomandibular or spiracular cleft). Only the myotomes and the cardiac wall show MyHC staining at this stage. The most anterior myotome has only its ventral half stained and is superior to the posterior half of the fourth pharyngeal arch.

By **stage 24** the posterior branchial arches are better defined compared to the previous stage and the fourth pharyngeal cleft is visible. The hyoid arch and branchial arches I and II show the first external gill buds that reach from their arch posteriorly over the adjacent cleft. The gill buds decrease in size from anterior to posterior (Fig. 3b). Dorsally between branchial arches I and II is a small anteroposteriorly orientated muscle, which is the ventral portion of an anterior developing myotome (Fig. 3b). Anteriorly on the hyoid arch and branchial arches I-III, faint MyHC staining can be detected indicating the initial differentiation of the dorsal constrictor muscles (*constrictor hyoideus* of the hyoid arch, and *constrictores branchiales I to III* of the branchial arches) (Fig. 3b). The staining decreases from anterior to posterior, which is undetectable in the mandibular arch. By **stage 26**, the fifth pharyngeal cleft is visible and the first myotome, which lies dorsally between branchial arches III and IV, is larger and more visible than in previous stages (Fig. 3c). The external gill branches on the hyoid arch and branchial arches I and II expand and the first gills appear on branchial arch III.

In **stage 27** the ventral portion of the most anterior differentiating myotome becomes visible dorsally between branchial arches I and II (Fig. 3d). The mandibular arch also shows an elongated thin muscle anlage anteroventrally located; this is the *intermandibularis* anlage, which is only separated by a small gap from the developing *interhyoideus* anlage of the hyoid arch (Fig. 3e). This *interhyoideus* anlage is continuous with the ventral portion of the *constrictor hyoideus*. The muscle anlagen of the dorsal constrictors extend and are more clearly visible (Fig. 3d, e): the hyoid arch includes the *constrictor hyoideus* anlage, while the branchial arches include the *constrictores branchiales I-III* (*constrictores branchiales superficialis*). These muscles are mainly dorsoventrally orientated, the *constrictor hyoideus* being the longest and stretching almost the entire length of the hyoid arch, and the *constrictor branchialis III* being the shortest, covering only half of the dorsoventral extension of the third branchial arch (Fig. 3d). The staining of the constrictors decreases from anterior (hyoid arch) to posterior (Fig. 3d) indicating the later development of more posterior muscles.

Major differences are then observed at **stage 28** (Fig. 4a-c). External gills appear on the mandibular arch and all posterior external gills are lengthened. Dorsal extraocular muscles are visible, the mandibular arch muscle anlage is expanded, and, from a lateral view, the *constrictor dorsalis* of this arch is visible just rostral to the spiracle (Fig. 4a). The most rostral portion of the mandibular muscles is the developing *preorbitalis*, caudally adjacent to the anlage of the *adductor mandibulae A2*; and caudomedially there is the *intermandibularis* anlage (Fig. 4b). At this stage, all mandibular arch muscles derive originally from a single elongated anlage that then separates during later stages into several regions (dorsal, middle and ventral), which then gives rise to one or more muscles. The hyoid arch muscle anlage also differentiates into three portions (Fig. 4a, b): the most rostro-dorsal one is the anlage of the *adductor arcus palatini*, the large lateral one is the anlage of the *constrictor hyoideus dorsalis*, and the most ventral portion is the *interhyoideus* anlage. The *adductor arcus palatini* develops from the most rostrodorsal portion of the *constrictor hyoideus* primordium; this rostrodorsal portion also gives rise to the *constrictor hyoideus dorsalis*. The *constrictores branchiales I-IV* stretch almost the entire dorsoventral length of their respective arches (Fig. 4a). The ventral branchial muscles *coracobranchiales* and the hypobranchial muscles *coracomandibularis* and *coracoarcualis* are distinguishable from all the other cephalic muscles because they develop from their region of insertion (i.e., from the mandible and branchial arches, respectively; compare Figs. 4b, f, 5b, d for *coracomandibularis* and Figs. 4f, 5b, d, 6c for *coracoarcualis*), while the mandibular, hyoid and branchial muscles develop from their region of origin, as do most cephalic muscles of other vertebrates (Fig. 4b; compare for examples Figs. 3e, 4f, 5d, 6c for the development of the *intermandibularis* and *interhyoideus*).

At **stage 29** the ventral extraocular muscles become visible (Fig. 4d). The differentiation of the mandibular muscles is now also noticeable from a lateral view (Fig. 4d). The dorsal constrictor separates into a dorsal part that extends posteriorly, the *spiracularis*, and a lateral portion, extending inferiorly and medially, the *levator arcus palatini* (Fig. 4d). The *preorbitalis*, *adductor mandibulae A2* and *intermandibularis* can be distinguished (Fig. 4d, f). The hyoid muscles *adductor arcus palatini* and *constrictor hyoideus dorsalis* start to separate, while ventrally the *interhyoideus* muscle grows (Fig. 4d, f). Within the branchial muscles only four *constrictores branchiales* – extending from lateral to ventral regions – are clearly visible (Fig. 4e, f). Ventrally the *coracobranchiales I-II* appear as faint stains at the base of the branchial arches I-II (Fig. 4f). Dorsal to branchial arches I and II, there is a faint anteroposteriorly orientated muscle staining, which indicates the initial differentiation of the *cucullaris* (Fig. 4e). The hypobranchial muscles *coracomandibularis* and *coracoarcualis* are now more distinguishable than in stage 28, and the hypobranchial muscle *sternohyoideus* is faintly visible just rostral to the *coracoarcualis* (Fig. 4f).

In **stage 30** the muscles described in stage 29 become more clearly separated (Fig. 5a), especially in ventral view (Fig. 5b). The main contrast to the former stage is that the *cucullaris* is now plainly visible as a thin band-like structure extending dorsally from branchial arch I to branchial arch IV (Fig. 5a). Ventrally the *sternohyoideus* becomes further distinguishable from the *coracoarcualis* (Fig. 5b). The *coracobranchiales I-III* can be seen ventrally to the respective branchial arches (Fig. 5b). In **stage 31** the lateral muscles of all arches continue to grow and differentiate without major changes relative to the previous stage (Fig. 5c), the *intermandibularis* and *interhyoideus* come into contact, and four *coracobranchiales* are now visible (Fig. 5d).

In **stage 32** all muscles are further differentiated (Fig. 6) and the head now appears, in a ventral view, almost completely covered by muscles (Fig. 6c). The *intermandibularis* and *interhyoideus* reach the ventral midline. The *coracobranchiales* are completely covered by the *constrictores branchiales*, and staining of deeper muscles is not visible. The *cucullaris* spans the entire length dorsally to the branchial arches (Fig. 6a, b). The cartilages are not clearly distinguishable because they are almost translucent at this stage with this methodology, but there was a faint attachment of the *cucullaris* onto the caudal branchial arch IV. Immediately caudal to the last branchial arch, the fibers of this muscle extend lateroventrally towards the pectoral girdle (Fig. 6a, b).

We observed neither the deeper true branchial muscles (*adductores branchiales*, *interbranchiales* and *interarcuales laterales*) nor the epibranchial muscles (i*nterpharyngobranchiales* and *subspinalis*); in adult sharks these deeper muscles are superficially covered by the *constrictores branchiales*, and attach onto branchial arches [18] (Table 1). That these muscles/bundles could not be seen can be explained by either the fact that they are not yet developed/differentiated or – more likely taking into account that all the other, more superficial, muscles of the branchial region are already seen – the penetration of the antibody was not deep enough. In fact, based on our studies of muscle development in other fishes and in tetrapods (cited above), it is very likely that most, or all, of these muscles started to differentiate in the oldest shark specimen(s) analyzed by us.

Thus, in summary, the anlagen (primordia) of mandibular arch muscles appear after the anlagen of hyoid arch muscles. The first branchial muscle anlagen can be observed simultaneously with the anlagen of the hyoid arch muscles, with the staining fainting from anterior to posterior (Fig. 3d). Still, analyzing the detailed appearance of muscles we could observe that muscles develop following an anterior to posterior direction, from the hyoid to the branchial arches and from outside to inside, i.e., lateral muscles develop before ventral muscles (except within the mandibular arch), and superficial muscles develop before deep muscles. Most muscles develop from their region of origin to their region of insertion (compare for examples Figs. 3e, 4f, 5d, 6c for the development of the *intermandibularis* and *interhyoideus*), except for the ventral branchial muscles *coracobranchiales* and the hypobranchial muscles *coracomandibularis* and *coracoarcualis*, which develop from their region of insertion to their region of origin (compare Figs. 4b, f, 5b, d for *coracomandibularis* and Figs. 4f, 5b, d, 6c for *coracoarcualis*).

## Discussion

### On the origin of the pectoral and pelvic musculatures

In chondrichthyan and osteichthyan fishes the pectoral appendages are invariably described as developing before the pelvic appendages, in contrast to the condition found in most tetrapods, where they develop relatively simultaneously [55, 56]. In *S. canicula* the pelvic fin indeed starts to develop later than the pectoral one: the first signs of a pectoral fin outgrowth were identified as early as stage 19, while pelvic fin development was only detected at stage 25 (Fig. 1). Moreover, as previously suggested [9], all events characterizing fish fin development, such as formation of a transient AER, conversion of this structure into an AEF, outgrowth and differentiation of endoskeleton elements, occur earlier in the pectoral fins than in the pelvic fins. Here we show that appendicular myogenesis also occurs earlier in the pectoral fins (stage 28) than in the pelvic fins (stage 29) in *S. canicula*. Interestingly, the formation of the *abductor* and *adductor* muscles in the pectoral fins occurred simultaneously with the formation of the preaxial muscle, the *pterygialis cranialis* (Table 1). All these muscles seem to have been present in the last common ancestor (LCA) of the crown-group Gnathostomata [6, 22, 57]. Therefore, the non-simultaneous development of pectoral and pelvic musculature, which is commonly observed in osteichthyan fishes, may reflect the ancestral developmental process in the gnathostome lineage.

In adult chondrichthyans the pectoral *abductor* and *adductor* and the pelvic *adductor* have superficial and deep bundles, but the pelvic *abductor* has instead proximal and distal bundles, demonstrating that the significant anatomical differences between the pectoral and pelvic appendages of sharks concern not only hard tissues, but also soft tissues such as muscles [22, 49, 58]. Our results demonstrate that during early developmental stages the muscles of the pectoral and pelvic fins are more similar to each other than in adulthood. While developing, there are mainly two major undivided muscles in each fin, *abductor* and *adductor*, except for a preaxial muscle present only in the pectoral fin (the *pterygialis cranialis*). Thus, during late development, most likely after hatching, pelvic muscles undergo further elaboration becoming rather distinct from pectoral muscles. Interestingly, while we could observe the formation of the *abductor* and *adductor* muscles in the pelvic fins up to stage 34, we could not detect the development of the *protractor* even in the oldest stages, which suggests that considerable development of the pelvic musculature occurs, in fact, after hatching.

Apart from the differences between the adult *abductors* of the pectoral and pelvic appendages, and between the time of appearance of the musculature of each of these appendages, there is another major difference between the musculature of these appendages in *S. canicula*: the presence of several muscles connecting the pectoral girdle to cranial elements, such as the *coracomandibularis*, *coracoarcualis*, *coracobranchiales*, and the *cucullaris,* which all develop from the head region to the pectoral girdle region and which have no corresponding muscles in the pelvic appendage. This latter difference stresses the point that there are major functional and evolutionary reasons for the spatial correlation of the pectoral girdle with the skull in early gnathostomes: the internal branchial chamber seems to restrict the development of the pectoral girdle more anteriorly, which forms a protection for the pericardial cavity and an insertion for the pectoral fins [58].

In fact, studies in chondrichthyans have shown that the formation of branchial arches in sharks and the tetrapod forelimb share strikingly similar developmental mechanisms [47, 48], somewhat consistent with the view that the branchial arches and the pectoral appendage might be highly related evolutionarily/developmentally see also [59]. For example, *sonic hedgehog* (*Shh*) is crucial to establish the anteroposterior polarity in both the outgrowing fin−/limb-bud and the developing gill arch [48]. Other studies analyzed the body wall formation in lampreys as compared to gnathostomes, which is relevant as the gnathostome paired appendages start as outgrowths of body wall somatopleure [60]. The somatopleure is a tissue containing somatic lateral plate mesoderm and overlying ectoderm [60]. Lampreys are cyclostomes, i.e., vertebrates without jaw and paired fins. Tulenko and colleagues [60] suggest that the somatopleure is eliminated in lampreys while the lateral plate mesoderm is separated from the ectoderm and isolated to the coelomic linings during myotome extension. One way to interpret those data is that the somatopleure may have originally persisted close to the gills, established a pectoral fin, and afterwards, spread posteriorly to the pelvic level [60]. This model has similarities to variations of the gill arch hypothesis [59]. Interestingly, a recent study identified a *Tbx5* fin enhancer, CNS12, in the noncoding region downstream of *Tbx5* locus [61]. The enhancer CNS12 was suggested to have driven the reporter gene expression in the lateral plate mesoderm posterior to the heart – a region where vertebrates with pectoral appendages show an apomorphic *Tbx5* expression pattern [61]. In the cephalochordate amphioxus *Tbx4/5* is expressed in the pharyngeal and posterior mesoderm together with cardiac genes and is relevant for the development of a noncentralized heart [62]. Other marker genes for vertebrate head and trunk mesoderm are also expressed in overlapping domains in amphioxus dorsal mesoderm, what indicates that the mesoderm is not yet differentiated along the craniocaudal axis [62]. These data thus support the hypothesis that the mesoderm of the posterior head region, the heart, and pectoral appendages might have originated from a common ancestral region. This scenario might be an example for deep homology, in which structures evolve by the modification of pre-existing genetic regulatory circuits established in early metazoans [63]. In fact, it was recently shown that the pharyngeal (head) muscles and the myocardium are developmentally and evolutionary more linked to each other than previously thought and that the so-called cardiopharyngeal field was likely present in the last common ancestor or of Olfactores (tunicates + vertebrates) [64].

The observations regarding the development of the *cucullaris* in *S. canicula* reinforce the idea of an ancestral close association between the head and pectoral girdle musculature. We showed that the anlage of the *cucullaris* clearly appears in the dorsal region of the branchial arches, without any connection between it and the anterior somites in early development (Fig. 4e). This further supports the hypothesis that the *cucullaris* is a true branchial muscle, as defended by classical authors such as Edgeworth [23] and in more recent developmental and molecular works [65]. *Tbx1* mutant mice, for example, have no *trapezius* or *sternocleidomastoideus*, which are derivatives of the *cucullaris*, and no branchial muscles, while somite-derived limb muscles are unaffected [66]. Further evidence was provided by a fate map study in *Ambystoma mexicanum* where it was shown that the lateral plate mesoderm contributes to posterior *branchial arch levators* and to the *cucullaris*, what led the authors to suggest that this mesoderm should be regarded as posterior cranial mesoderm [67].

There are further indications consistent with the idea that the musculature connecting the head to the pectoral girdle could have derived from, or co-opted, similar developmental mechanisms than those used by the musculature of the posterior pharyngeal arches. In fact, there are three groups of muscles that connect these appendages to head structures other than the *cucullaris*: the hypobranchial muscles *coracomandibularis* and *coracoarcualis* attach the pectoral girdle to the mandible and (mostly) to the ceratohyal, respectively, while the ventral branchial muscles *coracobranchiales* connect it to the branchial arches. The muscles *coracomandibularis* and *coracoarcualis* cannot be used to support a similarity between the pectoral and posterior pharyngeal arch musculatures, because they are hypobranchial muscles derived from somites, and not branchiomeric head muscles, as are most of the muscles that connect the posterior pharyngeal arches to other cranial structures. However, the presence of the true branchial muscles *coracobranchiales* connecting the pectoral girdle to the branchial arches, exactly as numerous branchial muscles connect the branchial arches to each other, might constitute an argument consistent with the idea of a deep association between the pectoral girdle musculature and the branchial arch musculature. Particularly because the pelvic musculature has of course no muscles at all connecting it to the head, and thus to any branchial arch. These data contradict the hypothesis that pectoral and pelvic appendages and associated soft tissues are strictly serial homologous because this does not refer merely to the different topological position of the pectoral vs. pelvic appendages. Instead, this refers to completely different types of tissues, derived from completely types of primordia, being part of each of these two types of appendages. That is, the pectoral appendage includes/is related to branchial muscles that are derived from the cardiopharyngeal field, while no such muscles are related/part of the pelvic appendage, which exclusively includes muscles derived from somites.

Authors have recently suggested that in some placoderm fossils the pectoral and pelvic appendages seem to be more similar than previously thought [36]. However, these studies do not include reconstructions of appendicular soft tissues such as muscles, which are crucial to discuss the similarity vs. dissimilarity of the pelvic and pectoral appendages as a whole. Anatomical and developmental studies performed on extant animals considered to have retained plesiomorphic musculature of gnathostomes [7], like chondrichthyans, show several aspects of dissimilarity between these appendages [18, 22]. Thus, strict serial homology does not seem to explain the origin of all the tissues that constitute/attach onto the pectoral and pelvic appendages. Further information on the cephalic and appendicular musculature of basal gnathostome lineages, such as the placoderms, would be ideal to infer to which extent chondrichthyans are plesiomorphic for this specific trait. We favor a scenario in which the different components of the pectoral and pelvic appendages may have arisen from distinct evolutionary processes leading to the integration of homoplastic structures. Moreover, the morphological evolution of the pectoral and pelvic appendages may have been conditioned by the position where they develop along the body axis, which result in distinct muscular phenotypes, including the crucial difference of the strong musculature connecting the pectoral girdle and the cranium.

### Myogenic progression during muscle development

Previous studies have suggested that various vertebrate groups share a temporal and spatial myogenic progression during the development of the cephalic muscles: from lateral/superficial to ventral/medial (outside-in), from origin to insertion, and from anterior to posterior [28, 32, 33]. Additionally, cephalic muscle differentiation seems to be tightly correlated with the development of cephalic cartilages, which was formerly described by various authors [68–71]. However, it was not previously addressed whether a similar myogenic progression is also detected in chondrichthyans. Our data reveal that, in *S. canicula,* the lateral muscles of one arch differentiate before the ventral muscles of the same arch. Thus, cephalic muscle development occurs following a lateral to ventral myogenic progression, which resemble the process described in osteichthyans such as zebrafish, lungfish, amphibians, and birds [28–33, 70, 72]. This pattern is clearer in the branchial arches than in the mandibular and hyoid arches, with the mandibular arch being the only exception: the ventral *intermandibularis* develops before the other muscles of the first arch, which are more lateral.

In both the head and paired appendages of *S. canicula*, muscles normally develop from their region of origin to their region of insertion, as was previously reported for the cephalic musculature of other osteichthyans [28, 29, 32, 33, 70]. The only exceptions, within the cephalic muscles analyzed by us, are the *coracomandibularis, coracoarcualis* and the *coracobranchiales*, which developed in the head region from their adult region of insertion (mandible, ceratohyal, and branchial arches, respectively) and then extend posteriorly during development towards their adult region of origin (pectoral girdle). Only a few other exceptions to this origin-insertion myogenic progression were formerly described [72]. Muscles with attachments on these cartilages remain without other attachments until the formation of the cartilages that lie in the adult region of origin of these muscles (e.g., otic capsule, pterygoid bone). This indicates that head muscle development depends on the underlying skeletal development [71], and this is probably why we see such a pattern in the *coracomandibulari*s, *coracoarcualis* and *coracobranchiales* of *S. canicula*, as the coracoid develops later than Meckel’s cartilage, ceratohyal, and the branchial cartilages.

As most previous studies describing an anterior to posterior myogenic progression of the cephalic muscles of various non-chondrichthyan taxa (Table 2) differ in their methodology, we used fiber development as a criterion to access and compare order of development, and we grouped all muscles of the same pharyngeal arch into a single group. By doing this, one can consistently compare the data obtained for each taxon, and detect their developmental progression. All embryonic/larval amphibians shown in Table 2 develop their mandibular and hyoid arch muscles simultaneously and in most species (13 out of 20) these muscles also develop simultaneously with the first branchial arch muscles. In the amniote groups Aves and Theria the mandibular arch muscles clearly develop earlier than the hyoid arch muscles, which even develop later than the muscles of branchial arch I in Theria (Table 2). In *S. canicula* the mandibular muscles, in contrast, develop after the hyoid muscles, while within the hyoid and branchial muscles the normal anteroposterior myogenic progression of muscle differentiation takes place. Furthermore, other branchial muscles develop following an anterior to posterior myogenic progression with the muscles associated to the last arch developing latest.

**Table 2:** Relative order of cephalic muscle development in selected vertebrates (based on first appearance of myofibers). Sources of developmental description are shown in the right column. Muscles in the same box develop simultaneously. However, it should be mentioned that if M, H, B, appear simultaneously, it is almost always because only the most anterior one or two arches develop simultaneous with the M and H. **M** – Mandibular arch muscles, **H** – Hyoid arch muscles, **B** – Branchial arch muscles, **Hy** – Hypobranchial arch muscles, **L** – Laryngeal muscles, **A** – extrinsic ocular muscles

Species/Order of appearance	1	2	3	4	5	6	Source
Non-tetrapods							
*Scyliorhinus canicula*	HB	MAHy					THIS STUDY
*Polypterus senegalus*	H	MA	Hy	B			Noda et al. [76]
*Danio rerio*	MAHy	H	B				Schilling and Kimmel [70]
*Neoceratodus forsteri*	MH	BHy	A				Ericsson et al. [73]
*N. forsteri*	H	M	B	Hy	L	A	Ziermann [32]
Tetrapods							
Urodela							
*Ambystoma mexicanum*	MHBHy	L	A				Ziermann [32]
*A. mexicanum*	MHB						Ericsson and Olsson [33]
*Ichthyotriton (Mesotriton) alpestris*	MH	BHy	A				Ziermann [32]
*Lissotriton vulgaris*	MHHy	BL	A				Ziermann [32]
*Necturus maculosus*	MHBHy						Platt [78]
Anura							
* Ascaphus truei*	MHBHy	A	L				Ziermann [32]
* Xenopus laevis*	MHA	BHy	L				Ziermann and Olsson [31]
*Hymenochirus boettgeri*	MH	BHyL	A				Ziermann [32]
*Discoglossus galganoi*	MH	BHy	LA				Ziermann [32]
*Discoglossus pictus*	MHBHyL	A					Ziermann [32]
*D. pictus*	MHB						Schlosser and Roth [79]
*Bombina orientalis*	MH	B					Ziermann [32]
*Bombina variegata*	MH	B	L	HyA			Ziermann [32]
*Pelodytes punctatus*	MHB	L	Hy	A			Ziermann [32]
*Pelobates fuscus*	MHB	HyL	A				Ziermann [32]
*Hyla cinerea*	MHB	HyL	A				Ziermann [32]
*Lepidobatrachus laevis*	MHBHyL	A					Ziermann [32]
*Bufo brongersmai*	MH	BHy L	A				Ziermann [32]
*Bufo speciosus*	MHB	L	Hy				Ziermann [32]
*Phrynomerus bifasciatus*	MHB	HyA	L				Ziermann [32]
*Kaloula pulchra*	MHB	HyL	A				Ziermann [32]
*Eleutherodactylus coqui*	MHBHy						Schlosser and Roth [80]
Aves							
* Gallus domesticus*	A	M	H	B			Noden et al. [81]
*Coturnix coturnix*	M	HB					McClearn and Noden [72]
Theria							
*Monodelphis domestica*	Hy	MB	HL	A			Smith [82]
*Mus musculus*	Hy	M	B	H			Kaufman and Kaufman [83]

However, one should note that in other fishes there are also exceptions to the anteroposterior myogenic progression (Table 2), which makes it difficult to infer whether this pattern is even the most commonly found in non-tetrapod vertebrates. For instance, two studies of the Australian lungfish (*Neoceratodus forsteri*) found minor differences in the developmental pattern of cephalic muscles [32, 73] and, while other developmental studies of lungfishes exist, none of them mentions the timing to clarify this pattern. In the zebrafish, the mandibular arch muscles develop before the hyoid arch muscles [70] and a recent study of the Longnose Gar (*Lepisosteus osseus;* Actinopterygii) describes a simultaneous development of mandibular and hyoid muscles [74]. The *Polypterus senegalus* belongs to the Polypteriformes, which is the most basal extant actinopterygian family [75], and was also described as developing the hyoid muscles before other cranial muscles [76].

What can be inferred from the developmental studies on gnathostome muscle development, summarized here, is that the hyoid arch muscles develop and differentiate before the branchial arch muscles in non-amniote vertebrates, as also described in the results presented here. The order of appearance of the mandibular arch muscles in vertebrates seems to be more variable (Table 2). Unfortunately, no study of agnathans explicitly states the order of development of each cephalic muscle, which is required in the future, to investigate which pattern is plesiomorphic, and to discuss its implications for our understanding of the evolution of the musculature in vertebrates and gnathostomes.

### Associations between ontogeny and phylogeny

Our previous works have indicated that in zebrafish and salamanders there is generally a parallelism between the order in which each cephalic muscle develops and the order in which each muscle was acquired during evolution (‘phylo-ontogenetic’ parallelism), barring only a few exceptions [28, 34]. A major problem with inferring a parallelism between the developmental order of appearance of muscles in sharks and the order in which the muscles appeared in evolution is that most of the muscles found in sharks and other gnathostomes are not present in any non-gnathostome extant taxon. Furthermore, there are insufficient detailed muscle reconstructions in fossils representing the transitions from agnathans to gnathostomes, making it difficult to infer the evolutionary order of appearance of the shark muscles.

However, given the phylogenetic position of sharks and their plesiomorphic muscular structure among gnathostomes [36], our observation that mandibular muscles develop after hyoid muscles implies that this might have been the ancestral process in the LCA of the crown-group Gnathostomata. In line with this idea, the simultaneous development of these two muscular units observed in osteichthyans may reflect derivation from the ancestral process. Interestingly, Miyashita [77] recently proposed that the ancestral mandibular arch was distinct from the pharyngeal arches, and only became secondarily similar to those structures during the evolutionary process that culminated with the origin of gnathostomes. Thus, mandibular and hyoid structures may have arisen from independent developmental processes, which then converge becoming increasingly similar over time. The patterns of cephalic muscle development observed in *S. canicula*, and particularly the fact that the mandibular muscles develop later than those of more posterior arches, breaking the seemingly stable anteroposterior myogenic progression seen in these latter arches in most osteichthyan clades, can thus provide important insights for further studies on the origin and early evolution of the gnathostome jaws.

## Conclusions

1. Our dissections and analysis of muscles in *S. canicula* are consistent with idea that there is an anatomical/functional association between the musculature associated with the pectoral girdle and that associated with the posterior branchial arches in the crown-group Gnathostomata. This contradicts the view that the pectoral and pelvic appendages are strict serial homologues in these animals. Instead, we favor a scenario in which the pectoral girdle musculature may have arisen from a non-homologous process to the one involved in the origin of the pelvic musculature.

2. In both the head and paired appendages of *S. canicula*, muscles normally develop from their region of origin to their region of insertion. The only exceptions within all the cephalic muscles are the *coracomandibularis, coracoarcualis,* and the *coracobranchiales*, which develop from their adult region of insertion (mandible, ceratohyal, and branchial arches, respectively), and then extend posteriorly towards their adult region of origin (pectoral girdle). Furthermore, during cephalic muscle development, a lateral to ventral pattern can be observed, with the mandibular arch being the only one where there is an exception with a ventral muscle developing before the lateral ones. In *S. canicula* the mandibular arch muscles develop later than the hyoid muscles, while among the hyoid and branchial muscles one can observe an anteroposterior myogenic progression. Even with the exceptions described here, cranial muscle development appears to be highly conserved in gnathostomes.

3. In the chondrichthyan species analyzed here, the mandibular muscles develop later than the hyoid muscles as was also described for *P. senegalus* [76], which is a member of the most basal extant actinopterygian group Polypteriformes [75]. In contrast, in most osteichthyans the mandibular muscles develop at the same time, or even earlier, then the hyoid muscles. A parallelism between ontogeny and phylogeny could be established if future studies provide further evidence consistent with Miyashita’s recent idea [77] that the mandibular arch was originally not integrated with or was not similar to the ancestral pharyngeal arches, and only became secondarily integrated with/similar to them in the transitions that lead to the LCA of crown-group Gnathostomata.
